# Extra-skeletal Ewing sarcoma in a 63-year-old female with a history of triple-negative breast cancer: a case report and literature review

**DOI:** 10.3389/fonc.2025.1545896

**Published:** 2025-05-23

**Authors:** Man Kit Ho, Golnesa Safavi, Won Jin Jeon, Evelyn Mendoza

**Affiliations:** ^1^ Department of Internal Medicine, Loma Linda University Medical Center – Murrieta, Murrieta, CA, United States; ^2^ Division of Medical Oncology and Hematology, Department of Internal Medicine, Loma Linda University, Loma Linda, CA, United States; ^3^ Division of Medical Oncology and Hematology, Department of Internal Medicine, Loma Linda University Medical Center – Murrieta, Murrieta, CA, United States

**Keywords:** extra-skeletal Ewing sarcoma, EWSR1-FLI1 fusion, EWSR1-ERG fusion, surgical resection, chemotherapy, radiation therapy

## Abstract

Ewing sarcoma (ES), particularly Extra-skeletal Ewing sarcoma (EES), is a rare, aggressive tumor predominantly affecting adolescents and young adults, yet it can occur in older patients, leading to misdiagnosis and delay in treatment. The standard approach includes surgical resection, chemotherapy, and radiation therapy for unresectable disease. This case report presents a 63-year-old female patient with a history of triple-negative breast cancer, who was discovered to have a soft tissue tumor in the left medial thigh. Initially misdiagnosed as rhabdomyosarcoma, the diagnosis of EES was ultimately confirmed via RNA sequencing revealing the EWSR1-FLI1 fusion gene. She underwent neoadjuvant chemotherapy followed by radical resection of the tumor.

## Introduction

Ewing sarcoma (ES) is a highly aggressive tumor predominantly affecting the bones of adolescents and young adults (1). Extra-skeletal Ewing sarcoma (EES) is a rare soft tissue malignant tumor belonging to the Ewing sarcoma family of tumors (ESFT) that originates from unique mesenchymal progenitor cells (1). EES is ten times less common than skeletal ES, with an incidence of 0.4 per million, representing less than 5% of all soft tissue sarcomas (2, 3). While 95% of ES cases occur between ages of 4 and 25, EES is more frequently diagnosed in older adults, often presenting with a female predominance (1, 2, 4). This report highlights a case of EES initially misdiagnosed as rhabdomyosarcoma, ultimately confirmed through RNA sequencing with positivity of EWSR1-FLI1 fusion.

## Case presentation

A 63-year-old woman presented to the oncology office in February 2024 with an enlarging mass in the left medial thigh. She reported no fever, bone pain, or joint pain. Her medical history included epilepsy and stage IIB triple-negative invasive ductal carcinoma of the right breast without gene mutation in 2020. She was treated with bilateral mastectomy with perioperative chemotherapy in 2021 including a total of 240 mg/m² of doxorubicin. Physical examination revealed a 5 x 5 cm subcutaneous, hard, and mobile mass in the left medial thigh.

An Ultrasound (US) in December 2023 indicated a markedly hypervascular solid lobulated 3.9 x 3.1 x 2.8 cm soft tissue mass in the mid inner left thigh, highly suspicious for malignancy tumor. In January 2024, a fine needle aspiration biopsy revealed malignant neoplasm without specific classification. A subsequent core biopsy suggested high-grade sarcoma with extensive necrosis, favoring rhabdomyosarcoma. However, RNA sequencing later confirmed EES with EWSR1::FLI1 fusion (**Box 1**). Further immunohistochemical (IHC) stains showed diffuse positivity for CD99 and 10% positivity for Ki67, while S100 protein and desmin were negative (**Box 2**).

Box 1 **Molecular study.**

**Table:** Cancer-type relevant biomarkers.

Biomarker	Method	Analyte	Result	Biomarker	Method	Analyte	Result
**EWSR1**	**Seq**	**RNA-Tumor**	**Pathogenic Fusion**	EZH2	Seq	DNA-Tumor	Mutation Not Detected
BRAF	Seq	DNA-Tumor	Mutation Not Detected	FUS	Seq	RNA-Tumor	Fusion Not Detected
MSI	Seq	DNA-Tumor	Stable	HRAS	Seq	DNA-Tumor	Mutation Not Detected
Mismatch Repair Status	IHC	Protein	Proficient (Intact)	IGF1R	CNA-Seq	DNA-Tumor	Amplification Not Detected
NTRK1/2/3	Seq	RNA-Tumor	Fusion Not Detected	KRAS	Seq	DNA-Tumor	Mutation Not Detected
RET	Seq	RNA-Tumor	Fusion Not Detected	MET	Seq	DNA-Tumor	Mutation Not Detected
Tumor Mutational Burden	Seq	DNA-Tumor	Low, 5 mut/Mb	MYOD1	Seq	DNA-Tumor	Mutation Not Detected
CDK4	CNA-Seq	DNA-Tumor	Amplification Not Detected	NF1	Seq	DNA-Tumor	Mutation Not Detected
ERBB2 (HER2/Neu)	Seq	DNA-Tumor	Mutation Not Detected		CNA-Seq	DNA-Tumor	Deletion Not Detected
PAX3	Seq	RNA-Tumor	Fusion Not Detected	NRAS	Seq	DNA-Tumor	Mutation Not Detected
PAX/	Seq	RNA-Tumor	Fusion Not Detected	PIK3CA	Seq	DNA-Tumor	Mutation Not Detected
PD-L1 (SP142)	IHC	Protein	Negative|0%	TP53	Seq	DNA-Tumor	Mutation Not Detected
PDGFRA	Seq	DNA-Tumor	Indeterminate		CNA-Seq	DNA-Tumor	Deletion Not Detected

**Table:** Genomic signatures.

Biomarker	Method	Analyte	Result
Microsatellite instability (MSI):	Seq	DNA-Tumor	Stable
Tumor Mutational Burden (TMB):	Seq	DNA-Tumor	Low (5)
Genomic Loss of Heterozygosity (LOH):	Seq	DNA-Tumor	Low - 5%

**Table:** Genes tested with pathogenic or likely pathogenic alternations.

Gene	Method	Analyte	Variant interpretation	Protein Alternation	Exon	DNA Alternation	Variant Frequency %
EWSR1	Seq	RNA-Tumor	Pathogenic Fusion	EWSR1-FLI1	/	–	–

Box 2 **Surgical pathology report of left inner thigh mass biopsy.**

**Initial Surgical Pathology Report.**

- HIGH-GRADE MALIGNANT NEOPLASM, FAVOR RHABDOMYOSARCOMA.
- EXTENSIVE NECROSIS PRESENT.

**Amended Surgical Pathology Report.**

- HIGH-GRADE SARCOMA, CONSISTENT WITH EXTRA-SKELETAL SARCOMA/PNET.
- EXTENSIVE NECROSIS PRESENT.

**Table:** Immunohistochemical Stain.

IHC Stain	Result	IHC Stain	Result
AE1/AE3	Negative	Ki67	10% Positive
S100 Protein	Negative	MyoD1	Focally positive
CD20	Negative	Myogenin	Negative
SMA	Negative	CD138	Negative
Desmin	Negative	CD99	Positive

**Figure 1:**
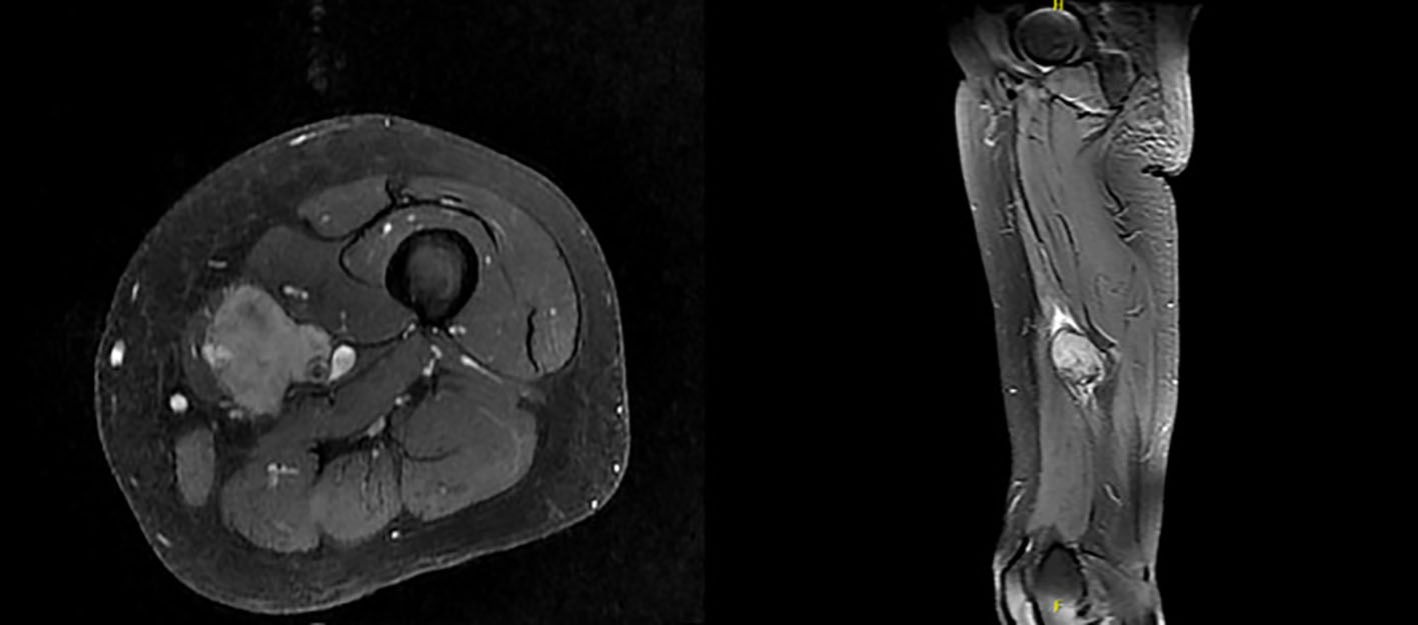
MRI of bilateral lower extremities with contrast in January 2024.

Magnetic Resonance Imaging (MRI) in January 2024 demonstrated a soft tissue mass measuring 4.3 x 3.2 x 6 cm, encasing the neurovascular bundle at the level of middle thigh, consistent with soft tissue sarcomatous mass (**Figure 1**). Fluorodeoxyglucose positron emission tomography (PDG-PET) indicated a hypermetabolic 5 cm mass in the left medial thigh without evidence of metastasis.

Neoadjuvant chemotherapy, comprising vincristine, doxorubicin, and cyclophosphamide, alternating with ifosfamide and etoposide, commenced in March 2024. Due to toxicity, the schedule was adjusted to every three weeks from every two weeks. Due to her prior treatment with doxorubicin for breast cancer, serial echocardiograms were conducted prior to each cycle of doxorubicin to monitor cardiac function. Additionally, she received dexrazoxane for cardioprotection prior to each dose of doxorubicin.

**Figure 2:**
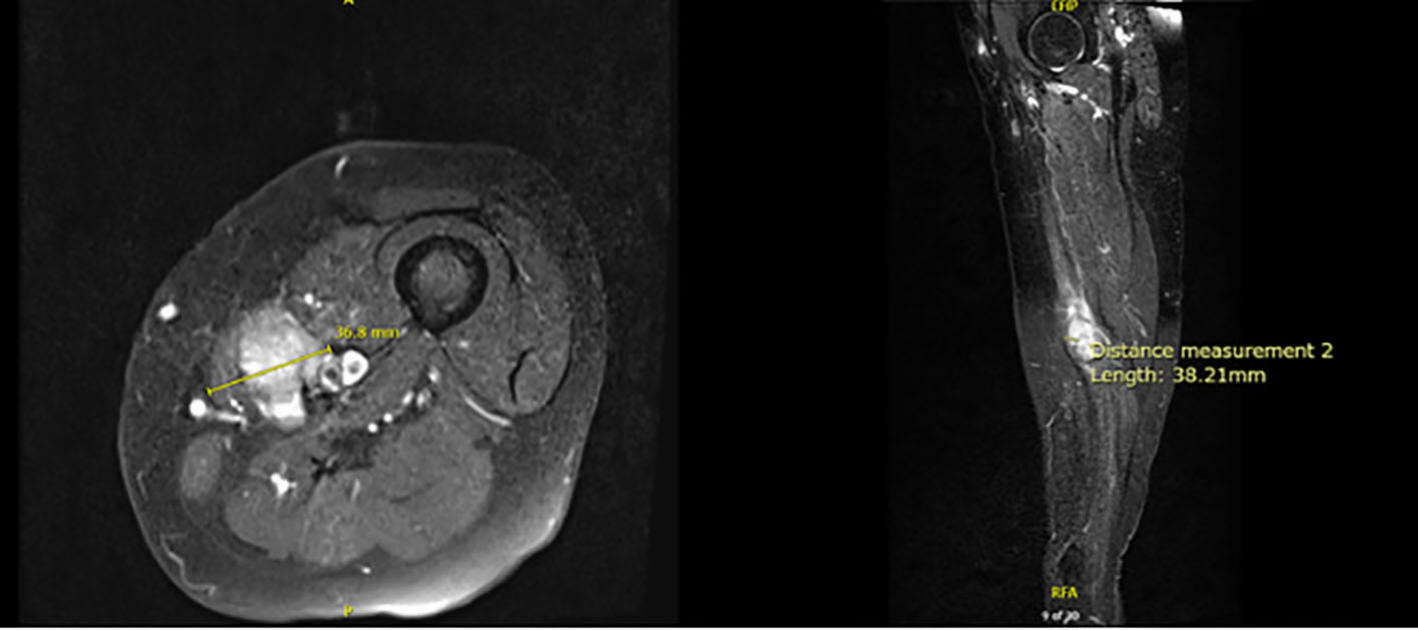
MRI of bilateral lower extremities with contrast in July 2024.

Following six cycles of neoadjuvant chemotherapy, MRI revealed a reduction in tumor size to 3.7 x 3.8 x 2.8 cm (**Figure 2**). Computed tomography (CT) Angiogram showed an avidly enhancing lesion within the left medial thigh measuring up to 2.8 cm with encasement/abutment of the femoral neurovascular bundle.

**Figure 3:**
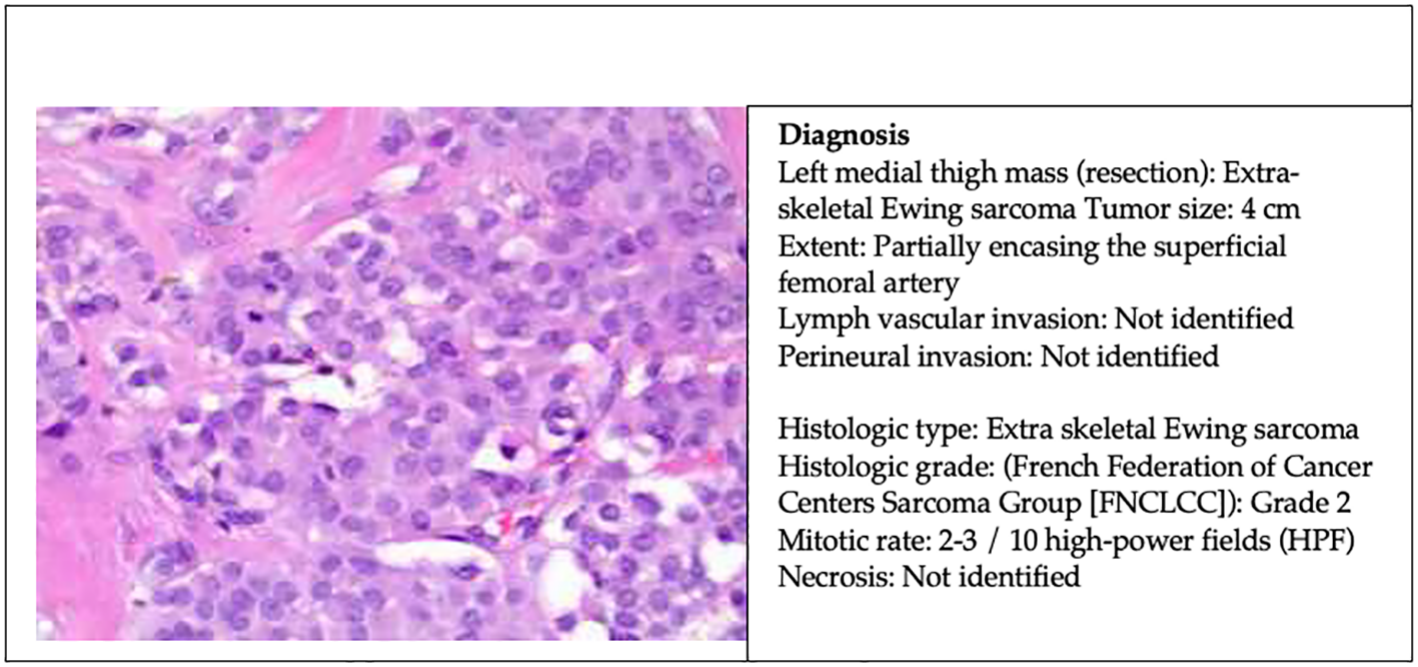
Surgical pathology report of left medial thigh mass resection.

After eight cycles of chemotherapy, the patient underwent radical resection of left medial thigh sarcoma with en-bloc segmental resection of involved sartorius, superficial femoral artery, and saphenous nerve. The pathology report revealed a 4 cm extra-skeletal Ewing sarcoma with clear margin and partially encasing the superficial femoral artery (**Figure 3**). The patient has been recovering well postoperatively. A PET-CT scan in 6 months was scheduled to monitor the disease activity. She will be continued with six additional cycles of adjuvant chemotherapy for a total of 14 cycles and followed up with medical oncology, surgical oncology, and vascular surgery within 2 weeks postoperatively.

## Discussion

EES is part of the ESFT and is much less common than skeletal ES. Approximately 85% of cases of ES are linked to the chromosomal translocation t(11; 22)(q24; q12), which creates the EWSR1-FLI1 fusion gene, functioning as both a pioneering transcription factor and a potent oncogene (5). About 10% of cases involve the translocation t(21;22)(q22;q12), leading to the EWSR1-ERG fusion protein, an oncogenic transcription factor that inhibits apoptosis (1, 6, 7). The remaining 5% exhibit rare translocations, including t(7;22)(p22;q12), t(17;22)(q12;q12), and t(2;22)(q33;q12) within EWSR1-ETS (Ewing sarcoma breakpoint region 1 - E-twenty six transformation specific) family, as well as t(2;16)(q35:p11) and t(16;21)(p11;q22) within the FUS-ETS (Fused in sarcoma - E-twenty six transformation specific) family (7). No studies have demonstrated a higher incidence of Ewing sarcoma in patients with breast cancer, or vise versa. However, research by Gorthi et al. revealed a link between BRCA1 and Ewing sarcoma. They found that EWS-FLI1 increases transcription to cause R-loops and block BRCA1 repair, which mimics BRCA deficiency without genetic mutation (8).

An observational study in pediatric and adult patients indicates that patients with EES tend to be significantly older than those with skeletal ES. EES most commonly occurs in the trunk (75.9%), followed by the head and neck (10.3%) and lower extremities (10.3%) (9). In contrast, a meta-analysis focusing on pediatric cases found no significant age association with EES incidence (10). Among children, the thorax is the most frequent location (33%), with extremities following closely (31%) (11). The lungs are the most common distant metastasis site in both pediatric and adult EES patients (9, 12). Overall, the prognosis of EES is generally more favorable than skeletal ES. Several factors, such as the presence of metastasis, older age, pelvic involvement, elevated white blood cell counts, elevated lactate dehydrogenase (LDH), low hemoglobin, large tumor size at diagnosis, and poor histologic response to preoperative chemotherapy are associated with worse outcomes (2, 13–15).

The diagnosis of ESS is often delayed due to its non-specific clinical presentation, typically manifesting as rapidly progressive swelling at the tumor site, often accompanied by compression-related symptoms (16). While US, CT, and MRI can help with diagnosis and local staging, their imaging characteristics are non-specific. Nuclear bone scans or PET scans are recommended for detecting bone metastases. Definitive diagnosis relies on pathological, immunohistochemical, and molecular findings (1, 2, 4, 17). Microscopically, EES typically consists of monomorphic small round blue cells with large spherical nuclei, inconspicuous nucleoli, and indistinct cytoplasmic borders (2, 4). CD99, expressed in about 95% of ES, is a sensitive but poorly specific diagnostic marker for ES as it is also expressed in other round cell sarcomas and even leukemias (4, 7); ZETB16 has demonstrated even greater sensitivity. Recently, NKX2.2 has been identified as a target of EWS-FLI1 (18) and shows high specificity when combined with CD99 or ZETB16 (19). Ewing cells also express FLI-1, CD45, S-100 protein, synaptophysin, vimentin, and desmin (1, 2, 20).

Given the sensitivity and specificity of IHC in ES, most of the ES can be diagnosed with pathology and IHC. However, for the cases with atypical clinical and pathological presentations, molecular genetics testing via Fluorescence *in-situ* hybridization (FISH), reverse transcriptase - polymerase chain reaction (RT-PCR), and/or direct sequencing becomes crucial for the diagnosis (2, 4, 21). A tumor sample can be analyzed for the EWS-FLI1 and EWS-ERG fusions using RT-PCR or for the EWS-associated fusions via FISH (22).

According to National Comprehensive Cancer Network (NCCN), all members of the ESFT, including EES, follow the same treatment algorithm. Wide surgical resection is recommended to enhance survival rates (23). NCCN advises at least nine weeks of multiagent chemotherapy prior to local therapy, which can improve five-year survival rate from 5-10% to over 65% by eliminating micrometastases (23, 24). VDC-IE (vincristine, doxorubicin, and cyclophosphamide alternating with ifosfamide and etoposide) is recommended as the first line systemic chemotherapy regimen (23). A randomized trial reported that VDC-IE given on an every-2-week schedule is more effective than an every-3-week regimen without increased toxicity (25). Large clinical trials indicate that 14 to 17 cycles of chemotherapy yield favorable outcomes, encompassing both neoadjuvant and adjuvant therapy (26).

Although EES is radiosensitive, surgical resection should be prioritized whenever feasible, as it has shown superiority over definitive radiotherapy. Perioperative radiotherapy may be considered for resectable lesions with positive margins, while definitive radiotherapy is reserved for inoperable lesions. Further, palliative radiotherapy can be beneficial for symptomatic patients in certain situations (14, 23). Metastatic disease or unresectable recurrent disease should be managed similarly as localized ES, utilizing systemic chemotherapy and local control strategies (2, 23).

New therapeutic approaches, particularly targeted therapy, are urgently needed to improve outcome for advanced disease. Despite the EWS-FLI1 fusion protein driving ES oncogenesis, inhibition of these proteins themselves has proved challenging and clinically unsuccessful. Instead, those targeted therapies acting on the tumor microenvironment appear more promising (27, 28).

## Conclusion

In this case report, our 63-year-old female patient was initially misdiagnosed with rhabdomyosarcoma in the lower extremity. However, RNA sequencing revealed the presence of the EWSR1-FLI1 fusion, confirming a diagnosis of EES, which necessitates a distinctly different treatment approach than that for rhabdomyosarcoma. Complicating her case further, the patient had previously received doxorubicin for triple-negative breast cancer, placing her at risk of exceeding the recommended maximum lifetime cumulative dose of 550 mg/m² (29). Despite the research indicates that Ewing sarcoma phenocopies BRCA1-deficient tumors, there is no study showed a direct connection between Ewing sarcoma and breast cancer. Given the significant mortality associated with metastatic EES, there remains an unmet need for biomarkers that can predict treatment response. Additionally, development of new therapeutic approaches, particularly targeted therapies, warrants further investigation to enhance the outcome for the patients with widely metastatic and refractory EES.

## Data Availability

The original contributions presented in the study are included in the article/supplementary material. Further inquiries can be directed to the corresponding author.
